# Characteristics of opioid prescribing to outpatients with chronic liver diseases: A call for action

**DOI:** 10.1371/journal.pone.0261377

**Published:** 2021-12-17

**Authors:** Olufunso M. Agbalajobi, Theresa Gmelin, Andrew M. Moon, Wheytnie Alexandre, Grace Zhang, Walid F. Gellad, Naudia Jonassaint, Shari S. Rogal

**Affiliations:** 1 Department of General Internal Medicine, University of Pittsburgh Medical Center, Pittsburgh, PA, United States of America; 2 Department of Biostatistics, University of Pittsburgh, Pittsburgh, PA, United States of America; 3 Division of Gastroenterology and Hepatology, University of North Carolina at Chapel Hill, Chapel Hill, NC, United States of America; 4 University of Pittsburgh School of Medicine, Pittsburgh, PA, United States of America; 5 Division of General Internal Medicine, University of Pittsburgh, Pittsburgh, PA, United States of America; 6 Center for Health Equity Research and Promotion, VA Pittsburgh Healthcare System, University Drive, Pittsburgh, PA, United States of America; 7 Division of Gastroenterology, Hepatology, and Nutrition, University of Pittsburgh, Pittsburgh, PA, United States of America; Auburn University, UNITED STATES

## Abstract

**Background:**

Chronic liver disease (CLD) is among the strongest risk factors for adverse prescription opioid-related events. Yet, the current prevalence and factors associated with high-risk opioid prescribing in patients with chronic liver disease (CLD) remain unclear, making it challenging to address opioid safety in this population. Therefore, we aimed to characterize opioid prescribing patterns among patients with CLD.

**Methods:**

This retrospective cohort study included patients with CLD identified at a single medical center and followed for one year from 10/1/2015-9/30/2016. Multivariable, multinomial regression was used identify the patient characteristics, including demographics, medical conditions, and liver-related factors, that were associated with opioid prescriptions and high-risk prescriptions (≥90mg morphine equivalents per day [MME/day] or co-prescribed with benzodiazepines).

**Results:**

Nearly half (47%) of 12,425 patients with CLD were prescribed opioids over a one-year period, with 17% of these receiving high-risk prescriptions. The baseline factors significantly associated with high-risk opioid prescriptions included female gender (adjusted incident rate ratio, AIRR = 1.32, 95% CI = 1.14–1.53), Medicaid insurance (AIRR = 1.68, 95% CI = 1.36–2.06), cirrhosis (AIRR = 1.22, 95% CI = 1.04–1.43) and baseline chronic pain (AIRR = 3.40, 95% CI = 2.94–4.01), depression (AIRR = 1.93, 95% CI = 1.60–2.32), anxiety (AIRR = 1.84, 95% CI = 1.53–2.22), substance use disorder (AIRR = 2.16, 95% CI = 1.67–2.79), and Charlson comorbidity score (AIRR = 1.27, 95% CI = 1.22–1.32). Non-alcoholic fatty liver disease was associated with decreased high-risk opioid prescriptions (AIRR = 0.56, 95% CI = 0.47–0.66).

**Conclusion:**

Opioid medications continue to be prescribed to nearly half of patients with CLD, despite efforts to curtail opioid prescribing due to known adverse events in this population.

## Introduction

Although the opioid prescribing rate in the United States has been declining since 2012 [1], in 2017, opioids accounted for 69% of drug overdose deaths, many of which are due to prescription opioids [2]. In fact, nearly half of patients starting treatment for opioid use disorder report their first exposure to opioids through an opioid prescription for pain [3]. Ongoing high rates of opioid prescribing are particularly problematic among high-risk subgroups, such as people with chronic liver diseases (CLD).

Frequent high-risk opioid prescriptions, defined as doses above 90 mg morphine equivalents (MME) or co-prescriptions with benzodiazepines, and opioid-related complications have been well-documented in patients with cirrhosis [4–11]. Moreover, chronic opioid prescriptions may present a barrier to liver transplantation, the only cure for cirrhosis [12]. Moreover, patients with cirrhosis have increased opioid toxicity compared to other populations [13]. These well-documented adverse consequences are concerning because opioid prescribing is extremely common for patients with cirrhosis. In fact, up to half of people with cirrhosis are prescribed an opioid each year [4].

While opioids are commonly prescribed to patients with cirrhosis, the degree to which this affects persons with less advanced CLD remains unclear. Yet, opioid prescribing is likely particularly problematic in this population. CLD itself is among the highest risk factors for opioid-related adverse events in the general population [14, 15]. Adverse consequences of opioids in this population include increased healthcare utilization [10]. Opioids have been associated with increased liver injury in murine models and in humans [16–22]. This may be in part due to opioid-related Inflammation and changes in the microbiome leading to accumulation of toxins [23–25]. Opioids are often co-prescribed with acetaminophen, risking unintentional liver injury for this population with underlying liver disease [4, 14]. Thus, to intervene to prevent adverse opioid-related outcomes in this population, it is critical to understand when in the course of CLD patients are prescribed opioids. Furthermore, with the rising burden of fatty liver disease, which affects one-third of the population in the US, it is increasingly important to understand how to safety manage pain in this growing population of persons with non-cirrhotic CLD [26–28].

Currently, because the risks and frequency of opioid prescribing to patients with CLD are unknown, it is challenging to combat this issue. Therefore, this retrospective cohort study aimed to investigate the medical and demographic characteristics associated with opioid prescribing to a population of outpatients with CLD and to further characterize high risk prescriptions, defined as defined as ≥90 MME per day or opioids prescribed with benzodiazepines [29]. We hypothesized the patients with non-cirrhotic CLD would have high rates of opioid prescriptions, similar to those with cirrhosis, suggesting that this issue starts earlier in disease progression than previously appreciated.

## Materials and methods

### Ethics approval

This study was approved by the University of Pittsburgh Institutional Review Board, protocol number PRO16120217, with a waiver of informed consent due to the retrospective nature of the study.

### Cohort definition

We identified a cohort of patients with CLD who had clinical encounters at the University of Pittsburgh Medical Center between October 1, 2015 and September 30, 2016. CLD was defined by the presence of one corresponding outpatient or inpatient ICD code for cirrhosis, alcohol-related liver disease (ALD), non-alcoholic fatty liver disease (NAFLD), viral hepatitis, hemochromatosis, autoimmune hepatitis, primary biliary cholangitis, or other CLD (S1 Table). Chart review was conducted for patients with codes for “unspecified viral hepatitis” to confirm that they had CLD. Patients were excluded if they were found to not have CLD, were not 18 years of age, or were pregnant. Each patient’s index visit was defined as the first outpatient, in-person visit between October 2015 and September 2016 with an ICD-10 code for CLD within the timeframe.

### Baseline characteristics

We extracted baseline demographic factors including age, sex, race/ethnicity (Hispanic/LatinX vs. non-Hispanic Black vs. non-Hispanic white vs. not specified vs. other specified race/ethnicity), and marital status. Insurance status (Medicaid vs. other) was used as a proxy for socioeconomic status, following examples from the literature [30, 31]. We defined comorbidities using two outpatient ICD-9 or ICD-10 codes within one year prior to the index visit and used previously published ICD coding strategies to define Charlson Comorbidity Index [32], mental health (i.e., anxiety and depression), alcohol use disorder, substance use disorders (SUDs), pain-related diagnoses [4], and hepatocellular carcinoma (ICD-10 C22.0, C22.9 and Z85.05). Etiology of liver disease was re-categorized as NAFLD vs. other since this was the most common etiology. Cirrhosis was defined by either an ICD-10 code at baseline for cirrhosis, a recognized complication of cirrhosis, or fibrosis-4 (FIB-4) score >3.25 [33].

### Opioid prescribing

Starting with an index clinical encounter, patients were followed for one year to assess opioid prescribing. All prescribed opioids (e.g., tramadol, morphine, fentanyl) were included in the definition of opioids, with the exception of cough syrups or medications used for opioid use disorder (e.g., buprenorphine), following previously-published methods and conventions [4, 34, 35]. We collected the dose and frequency of opioids and calculated milligrams of morphine equivalents using an “as-prescribed” approach, which assumes that patients take their prescribed opioids at the maximum dose and on the schedule recommended by their clinicians [36]. Patients were divided into three groups based on those prescribed no opioids vs. moderate-risk opioids vs. high-risk opioids. High-risk prescriptions were defined as an average dose ≥90 morphine milligram equivalents (MME) per day or co-prescription with a benzodiazepine, based on the Centers for Disease Control and Prevention guidelines [29]. We defined “moderate-risk” opioid prescriptions as <90 MME/day and with no benzodiazepine prescription.

### Analysis

Data were summarized using descriptive statistics, describing baseline covariates for the entire cohort, and then for patients with no opioid use, moderate-risk opioid use, and high-risk opioid use. Differences between groups were assessed using chi-square tests for categorical variables, Fisher’s exact test for sparse categorical variables (n<5), and ANOVA for comparison of groups greater than two, in order to assess the factors significantly associated with opioid status in univariate analyses. We subsequently assessed the factors associated with opioid prescriptions within one year after the index visit using multivariable multinomial regression with automated AIC optimization with the R MASS package [37].

## Results

### Baseline cohort characteristics

**Fig 1:**
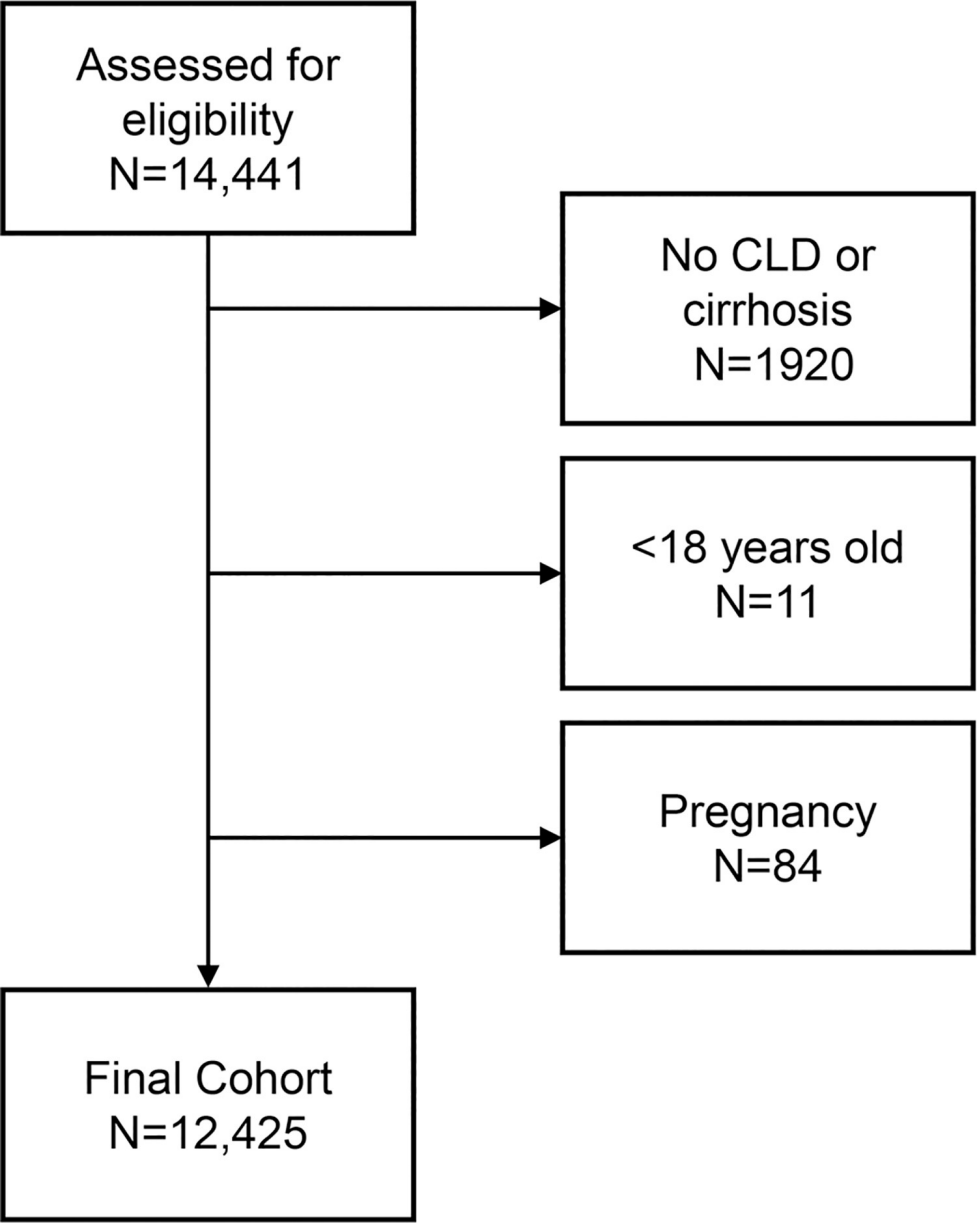
Cohort of patients with chronic liver disease.

After excluding 108 patients for pregnancy (n = 84), being under 18 years old (n = 11), and having no CLD (n = 1,920), the final cohort included 12,425 patients with CLD, including 3,980 with cirrhosis (Fig 1). The mean age was 58±13 and 51% were women. The most common etiology of liver disease was NAFLD (34%) (Table 1).

**Table 1:** Baseline characteristics of 12,425 patients with chronic liver disease, overall and by opioid prescriptions over follow-up.

Patient Characteristics	Total Cohort (N = 12,425)	No opioids (N = 6,625)	Moderate-risk opioid (N = 4,809)	High-risk opioid (N = 991)	P value
**Demographics**					
**Age (mean, sd)**	58±13	57±14	59±12	58±12	<0.001
**Female (n,%)**	6,357 (51)	3,195 (48)	2,582 (54)	581 (59)	<0.001
**Medicaid (n,%)**	1,456 (12)	654 (10)	634 (13)	168 (17)	<0.001
**Race/ethnicity (n,%)**					<0.001
** Hispanic/LatinX**	96 (1)	51 (1)	39 (1)	6 (1)	
** Non-Hispanic Black**	781 (6)	349 (5)	382 (8)	50 (5)	
** Non-Hispanic White**	11051 (89)	5854 (88)	4277 (89)	920 (91)	
** Not specified**	334 (3)	258 (4)	64 (1)	12 (5)	
** Other race/ethnicity**	163 (1)	113 (2)	47 (1)	3 (0)	
**Married (n,%)**	7,138 (58)	3,890 (59)	2,716 (56)	532 (54)	0.003
**Medical diagnoses**					
**Cirrhosis (n,%)**	3,980 (32)	2,064 (31)	1,557 (33)	359 (36)	0.005
**HCC (n,%)**	103 (1)	37 (0.6)	54 (1)	12 (1)	0.002
**NAFLD (n,%)**	4,190 (34)	2,307 (35)	1,627 (34)	256 (26)	<0.001
**Chronic Pain (n,%)**	5,420 (44)	2,135 (32)	2,637 (55)	648 (65)	<0.001
**Depression (n,%)**	1,887 (15)	667 (10)	950 (20)	270 (27)	<0.001
**Anxiety (n,%)**	1,690 (14)	683 (10)	753 (16)	254 (26)	<0.001
**Substance Use Disorder (n,%)**	826 (7)	297 (4)	412 (9)	117 (12)	<0.001
**Alcohol use disorder**	576 (5)	308 (5)	229 (5)	39 (4)	0.53
**Charlson Index (median, IQR)**	0 (0,2)	0 (0,1)	1 (0,2)	1 (0, 3)	<0.001

Abbreviations: HCC, hepatocellular carcinoma; IQR, interquartile range; NAFLD, nonalcoholic fatty liver disease.

High risk opioids are defined as >90MME/day or co-prescription with benzodiazepines; moderate risk are all other prescriptions opioids.

### Baseline characteristics associated with opioid prescriptions

After one year of follow-up, 47% of patients had been prescribed an opioid medication. Of those patients who were prescribed opioids, 17% received high-risk prescriptions, defined as ≥90 MME per day or opioids prescribed with benzodiazepines. Factors associated with high-risk opioid prescriptions in bivariate analyses included being female, unmarried, or white and having Medicaid insurance, cirrhosis, non-NAFLD-related CLD and chronic pain, depression, and SUDs (Table 1).

In the multivariate model, baseline factors significantly associated with “moderate-risk” opioid prescriptions included female sex (Adjusted Incidence Rate Ratio [AIRR] = 1.12, 95% CI = 1.03–1.21), Medicaid insurance (AIRR = 1.41, 95% CI = 1.24–1.60), baseline chronic pain (AIRR = 2.24, 95% CI = 2.07–2.43), depression (AIRR = 1.63, 95% CI = 1.45–1.84), SUDs (AIRR = 1.66, 95% CI = 1.41–1.96), and Charlson comorbidity score (AIRR = 1.17, 95% CI = 1.14–1.20). Factors associated with reduced risk of receiving moderate-risk opioid prescriptions included NAFLD (AIRR = 0.90, 95% CI = 0.82–0.98) and having unspecified race (AIRR 0.44, CI = 0.26–0.74) (Table 2).

**Table 2:** Final multivariate multinomial regression model of baseline factors associated with opioid prescriptions*.

Patient Characteristics	Moderate risk opioids			High risk opioids		
Demographics	IRR	CI		IRR	CI	
**Age**	1.00	1.00	1.00	0.99*	0.98	1.00
**Female**	1.12*	1.03	1.21	1.32*	1.14 1.53	
**Medicaid**	1.41*	1.24	1.60	1.68*	1.36	2.06
**Race/ethnicity (vs. white)**						
** Hispanic/LatinX**	1.29	0.81	2.05	1.05	0.42	2.67
** Non-Hispanic**						
** Black**	1.02	0.66	1.58	1.56	0.65	3.76
** Unspecified**	0.44*	0.26	0.74	0.69	0.24	1.98
** Other specified**	0.64	0.37	1.13	0.30	0.07	1.30
**Medical Conditions**						
**Cirrhosis**	1.04	0.95	1.14	1.22*	1.04	1.43
**NAFLD**	0.90*	0.82	0.98	0.58*	0.49	0.68
**HCC**	1.69*	1.08	2.63	1.69	0.85	3.38
**Substance use disorder**	1.66*	1.41	1.96	2.00*	1.56	2.56
**Chronic pain**	2.24*	2.07	2.43	3.40*	2.92	3.94
**Depression**	1.63*	1.45	1.84	1.87*	1.56	2.24
**Anxiety**	1.16*	1.03	1.31	1.85*	1.54	2.21
**Charlson index**	1.17*	1.14	1.20	1.27*	1.22	1.32

*Indicates statistically significant association, p<0.05.

High risk opioids are defined as >90MME/day or co-prescription with benzodiazepines; moderate risk are all other prescriptions opioids.

Abbreviations: IRR, incidence rate ratio; CI, confidence interval; NAFLD, nonalcoholic fatty liver disease; HCC, hepatocellular carcinoma.

The baseline factors significantly associated with high-risk opioid prescriptions (Table 2) included female gender (AIRR = 1.32, 95% CI = 1.14–1.53), Medicaid insurance (AIRR = 1.68, 95% CI = 1.36–2.06), cirrhosis (AIRR = 1.22, 95% CI = 1.04–1.43) and baseline chronic pain (AIRR = 3.40, 95% CI = 2.94–4.01), depression (AIRR = 1.93, 95% CI = 1.60–2.32), anxiety (AIRR = 1.84, 95% CI = 1.53–2.22), SUDs (AIRR = 2.16, 95% CI = 1.67–2.79), and Charlson comorbidity score (AIRR = 1.27, 95% CI = 1.22–1.32). NAFLD (AIRR = 0.56, 95% CI = 0.47–0.66) was associated with reduced risk of receiving high-risk opioid prescriptions.

## Discussion

Despite a reduction in opioid prescribing in the general US population, prescribing to individuals with CLD remains high. In this cohort of patients with CLD, nearly half of patients received opioid prescriptions in a single year, with nearly one fifth of those prescriptions being high-risk, despite extensive national opioid safety efforts [38]. High-risk opioid prescribing was more common to patients with CLD who were women and those with Medicaid insurance, depression, chronic pain, SUD, and higher comorbidity scores. This study expands upon work finding high rates of opioid prescribing to patients with cirrhosis by evaluating outpatients with all stages and types of CLD, suggesting that efforts to curtail opioid use for pain management should be considered earlier in the natural history of liver disease.

It is notable that rates of opioid prescribing were high among individuals *without* cirrhosis, suggesting that opioid prescribing starts early in the course of liver disease and must be addressed early. Other cohort studies have examined Veterans with cirrhosis, inpatients with decompensated cirrhosis, exclusively one etiology of CLD (e.g., hepatitis C), or cohorts established prior to efforts to curtail opioid use in the general population [9, 39–42]. This study is unique in including a general population with a diversity of disease etiologies and stages. The finding that opioid prescribing starts in this population prior to disease progression suggests a unique target for opioid de-implementation. Once individuals begin chronic opioids for chronic pain, it becomes more challenging to stop, or deprescribe, these medications [43, 44]. Deprescribing requires a careful, personalized approach [45]. Therefore, finding alternative solutions to manage pain early in the course of CLD may be key to avoiding initiation of opioids.

There are several challenges with analgesia that are unique to populations with CLD that may explain the high rates of opioid prescribing. In addition to contraindications to common analgesics, patients with CLD commonly have chronic pain, and this pain is often complex and complicated by prior SUDs and AUD, There is confusion, even among experienced clinicians, about the safety of the over-the-counter medications in this population [40, 46, 47]. Non-steroidal anti-inflammatory drugs (NSAIDs) are contraindicated, specifically in patients with cirrhosis, due to potential nephrotoxicity, exacerbations of ascites, and increased bleeding risk [47]. However, they are often prescribed due to the misperception that NSAIDs are safer than acetaminophen in this population. In fact, up to 2 grams of acetaminophen is allowable and first-line in this population, though higher doses can lead to hepatotoxicity [39, 46, 47]. Other potential opioid alternatives may include topical preparations (e.g., capsaicin) or local treatments (e.g., joint injections). However, perhaps the most effective and underutilized treatments are behavioral in nature.

Efforts to curtail opioid prescriptions are due to the key role that opioids to play in overdose-related deaths and the high numbers of opioid-related adverse events in general populations [35, 48, 49]. In fact, opioid-related deaths have contributed to a declining life expectancy in the US [50]. Moreover, opioid prescriptions are not effective for the treatment of chronic pain [51, 52]. As such, opioid prescribing has been a priority for US governmental agencies and regulatory bodies, resulting in a general decline in overall opioid prescribing over the last 10 years [53]. This reduction in prescribing in the general population highlights the striking nature of the ongoing crisis for patients with CLD, a population with a number of risk factors for increased adverse events from opioids.

Within this cohort of patients with CLD, high-risk opioid prescribing was more common among patients who had concurrent anxiety, depression, and SUDs, similar to prior findings in patients with cirrhosis [42]. In the general population, long-term prescriptions are also more common in these subpopulations, despite increased risk of medication-related overdose death in patients with mental health and substance use disorders [38, 54]. Because prescription opioids for chronic, non-malignant conditions are associated with increased depressive symptoms among individuals free of depression upon opioid treatment initiation, it is also concerning that opioids could worsen pre-existing mental health concerns [55]. Patients with cirrhosis were more likely to receive high-risk, but not any, opioids, even though this group is more likely to have complications from opioids vs. those with less advanced CLD. Thus, the patients most likely to receive higher risk prescriptions are also those at the highest risk of adverse opioid-related events.

Given the challenges of pain management in patients with liver disease, the strong associations between pain, substance use, and psychiatric symptoms in this population, and emerging evidence that opioids are not effective in treating chronic pain, a more comprehensive approach to pain and symptom management in this population is needed [42, 52]. It therefore is reasonable to pursue therapeutic options that address other possible underlying etiologies of pain, as well as offer a multidisciplinary and multidimensional treatment approach, including physical, behavioral, procedural, and pharmacologic interventions. Alternative analgesia may include topical preparations (e.g., capsaicin), which have a lower potential for systemic side effects. It is sometimes appropriate to prescribe opioids, after engaging in a careful shared decision-making process with patients. For example, at the end of life, when pain management is prioritized and short-term analgesia is needed, opioids are a reasonable option, even in high-risk patients. When opioids are prescribed, the safest formulations include hydromorphone and oxycodone *without* acetaminophen [13, 39, 46]. While tramadol is perceived to be safer and is frequently used, it is indeed more unsafe due to its first and second pass hepatic metabolism causing unpredictable metabolism [4, 13, 39, 46]. Providers prescribing opioids to patients with cirrhosis should follow general opioid safety guidelines, evaluate and follow for HE and consider prophylactic lactulose [5, 38]. In addition, clinicians should monitor the use of prescription opioids in individuals with mental health or SUDs, as well as screen for depression and SUDs when on opioid medications [38].

Despite the important finding that high rates of opioid prescribing and high-risk opioid prescribing persist in patients with CLD, there were notable limitations of this study. This was a single-center study of mostly white patients, precluding our ability to assess racial disparities in detail. Furthermore, comorbidities and psychiatric diagnoses were based on retrospective code-based determinations and not standardized, prospective instruments. The retrospective nature of the study also precluded our ability to discern the indication for the opioids, given the lack of sensitivity and specificity of ICD codes for this purpose (e.g., only 60% carried a diagnostic code for a painful condition). Opioids are often co-prescribed with acetaminophen, which has been previously assessed; however, this study was not designed to evaluate specific formulations of opioids [4, 56]. Limitations include the lack of clarity around indication for opioids in this population; this is a limit of most electronic health record studies in this area. We also used a conservative of definition of high-risk, including very high doses and benzodiazepines, while the “safer” doses of opioids in this population are likely much lower. Regardless of these limitations, these findings point to the importance of considering opioid-sparing pain management strategies for patients with cirrhosis and earlier stages of CLD.

## Conclusion

In this retrospective study, prescription opioid use was common in patients with CLD over a one-year period. Future research should evaluate the benefits and risks of alternative opioid-sparing and non-pharmacologic analgesia in this population.

## Supporting information

S1 Table ICD-9 and ICD-10 codes for chronic liver disease and decompensation. Abbreviations: CLD, chronic liver disease.
(DOCX)

## Abbreviations

CLD: chronic liver disease
HE: hepatic encephalopathy
ALD: alcohol-related liver disease
NAFLD: non-alcoholic fatty liver disease
SUD: substance use disorder
FIB-4: fibrosis-4
MME: morphine milligram equivalents
AIRR: Adjusted Incidence Rate Ratio
