# Supplementary material for: Characteristics of opioid prescribing to outpatients with chronic liver diseases: A call for action
Source: PLoS One. 2021 Dec 17;16(12):e0261377. doi: 10.1371/journal.pone.0261377 (PMC8682904; doi:10.1371/journal.pone.0261377)
Supplement: S1 Table — Abbreviations: CLD, chronic liver disease. (DOCX) [file pone.0261377.s001.docx]

|  | **ICD-9 Codes (prior to 10/1/15)** | **ICD-10 Codes** |
| --- | --- | --- |
| **CLD Conditions** | | |
| Cirrhosis | 571.2, 571.5, 456.0, 456.20, 456.1, 456.21, 789.5, 567.23, 572.2, 572.4 | K70.3x, K72.10, K74.60, K74.69, K76.6 |
| Alcoholic Liver Disease | 291.81, 291.1, 291.8, 291.9, 303.00, 577.0, 357.0, 425.5, 980.9, 305.00, 571.0, 571.1, 571.2, 571.3 | K70.3, K70.31 |
| Non-alcoholic Fatty Liver Disease | 571.8 | K75.81, K76.0 |
| Viral Hepatitis/Chronic Hepatitis | 070.44; 070.41; 070.51; 070.54; 070.71, 070.7, 070.44; 070.41; 070.51; 070.54; 070.71, 070.7, | B18.0, B18.2, B19.10, B19.11, B19.2, B19.21, K73.0, K73.2, K73.8, K73.9 |
| Hemochromatosis | 275.01 | E83.110 |
| Autoimmune Hepatitis | 571.42 | K75.81 |
| Primary Biliary Cirrhosis | 571.6 | K74.3, K74.5 |
| Other CLD |  | K76.89 |
| **Decompensation Event** | | |
| Ascites | 789.5, 789.59 | K70.11, K70.31, K71.51, K65.2, R18.8, K74.60 (plus “cirrhosis of liver with ascites”) |
| Hepatic Encephalopathy | 572.2, 070.44 | K72.91, K71.11, K72.11, K72.01, G93.40 |
| Variceal Bleeding | 456.20, 456.0 | I85.00, I85.01, I85.11, K70.30 (plus “bleeding esophageal varices in alcoholic cirrhosis”) |
| Hepatorenal Syndrome | 572.4 | K76.7 |
